# JamCatcher: A Mobile Jammer Localization Scheme for Advanced Metering Infrastructure in Smart Grid

**DOI:** 10.3390/s19040909

**Published:** 2019-02-21

**Authors:** Taimin Zhang, Xiaoyu Ji, Zhou Zhuang, Wenyuan Xu

**Affiliations:** School of Electrical Engineering, Zhejiang University, Hangzhou 310027, China; zhangtaimin@zju.edu.cn (T.Z.); zhuangzhou@zju.edu.cn (Z.Z.); wyxu@zju.edu.cn (W.X.)

**Keywords:** smart grid, advanced metering infrastructure (AMI), smart meters, jamming, radio interference, localization

## Abstract

As the core component of the smart grid, advanced metering infrastructure (AMI) is responsible for automated billing, demand response, load forecasting, management, etc. The jamming attack poses a serious threat to the AMI communication networks, especially the neighborhood area network where wireless technologies are widely adopted to connect a tremendous amount of smart meters. An attacker can easily build a jammer using a software-defined radio and jam the wireless communications between smart meters and local controllers, causing failures of on-line monitoring and state estimation. Accurate jammer localization is the first step for defending AMIs against jamming attacks. In this paper, we propose JamCatcher, a mobile jammer localization scheme for defending the AMI. Unlike existing jammer localization schemes, which only consider stationary jammers and usually require a high density of anchor nodes, the proposed scheme utilizes a tracker and can localize a mobile jammer with sparse anchor nodes. The time delay of data transmission is also considered, and the jammer localization process is divided into two stages, i.e., far-field chasing stage and near-field capturing stage. Different localization algorithms are developed for each stage. The proposed method has been tested with data from both simulation and real-world experiment. The results demonstrate that JamCatcher outperforms existing jammer localization algorithms with a limited number of anchor nodes in the AMI scenario.

## 1. Introduction

Advanced metering infrastructure (AMI) is the core component of the smart grid [1]. It enables bidirectional communication between utility companies and customers by integrating advanced sensors, smart meters, monitoring systems, computer software/hardware, and data management systems [2]. AMI plays a pivotal role in several critical tasks such as automated billing, demand response, load forecast, and management [3]. Governments around the world are putting effort into developing AMIs. For instance, a total of 65 million smart meters were projected to be installed in the U.S. by 2015, covering more than half of all U.S. households. China has installed over 400 million smart meters through its state-run utilities since launching the program in 2012. The U.K. government plans to roll-out smart meters in every household by 2020.

**Figure 1:**
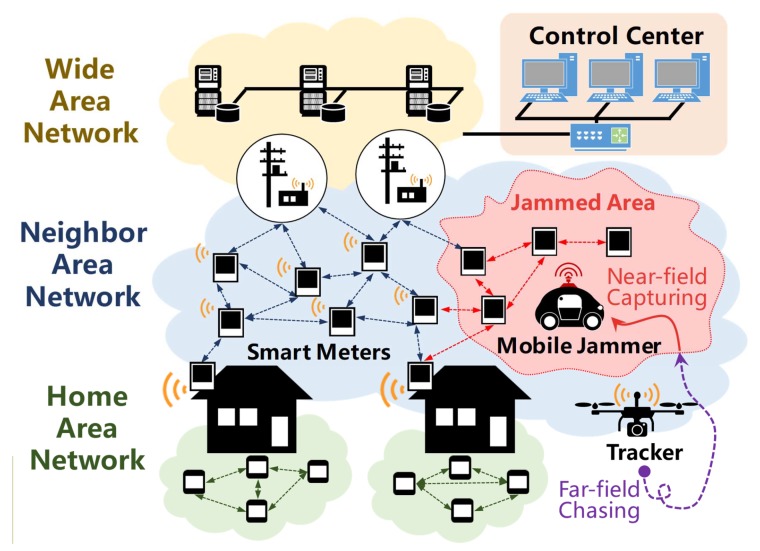
A jamming attack scenario in the neighbor area network of an AMI.

An AMI typically consists of smart meters in customers’ home area, a meter data management system (MDMS) at the control center, and communication networks at different levels of the infrastructure hierarchy that transmit data between smart meters and the MDMS. The tremendous amount of smart meters is connected through the neighborhood area network (NAN). As wireless communication technologies are usually utilized to create wireless multi-hop routes for smart meters, the NAN of AMI is vulnerable to jamming attack. A malicious attacker may use a jammer to interfere with the communication between smart meters (shown in Figure 1) and move in the NAN to affect as many smart meters as possible. The jamming attack could be the first step to launch a variety of crimes in the smart grid. Taking energy theft for example, the criminal could jam the power price signaling in a highly-populated city to manipulate the price and make a profit [4,5,6]. Furthermore, an adversary can launch a false data injection attack against the smart grid by jamming the wireless transmission between AMI sensors and a remote estimator, and simultaneously modify the system’s physical state to produce a misleading operational decision of the smart grid [7,8,9]. As AMI plays an important role in the smart grid, it is essential to defend AMI from such jamming attacks.

To defend the AMI communication networks against jamming attacks and further catch the jammers, accurate jammer localization is a key step. As industrial unmanned aerial vehicles (UAVs) have been a trend in smart grids, such as the application of high-voltage power lines’ inspection and power network damage assessment [10,11,12,13], a promising solution is to deploy a tracker (a drone or a mobile robot) in an AMI network to track the jammer without human intervention. The UAV-assisted tracker can move in the area where jamming attacks happened and localize the jammer with the help of affected smart meters. The tracker can collect measurements from smart meters directly, thus achieving real-time localization and tracking. In this paper, we present JamCatcher, a mobile jammer localization and tracking scheme for AMI networks. JamCatcher utilizes a tracker, e.g., a UAV, to measure the distance between each anchor node (i.e., smart meters) and the jammer with jamming signal strength (JSS) as the input, to finally derive the location of the jammer.

In developing JamCatcher, the main challenge is to localize the jammer with a limited number of JSS measurements from anchor nodes. This is due to two reasons, i.e., the sparse distribution of smart meters and the lossy link for data transmission. A sparse distribution means smart meters are far from each other. Taking a typical neighborhood in the U.S. as an example, smart meters are often tens of meters away from each other. As JSS decreases rapidly as a function of the distance, the estimated distance between the anchor node and the jammer becomes more inaccurate as the distance between them increases [14,15]. For instance, the results in [15] show the averaged estimation error increases from less than 1 m to 2.6 m as the distance increases from 10 m to 43 m. Thus, for good accuracy, only measurements from smart meters located within a specific range (referred to as effective range in the rest of the paper) of the jammer should be used, which limits the number of measurements. The lossy link means the links between the smart meters are characterized by high loss rates and instability due to the jamming attack, and the tracker can only receive part of the measurements sent by smart meters located in the effective range. Most existing jammer localization methods are not well applicable to AMI networks since they require anchor nodes to be densely distributed to achieve good accuracy [16,17,18,19,20,21,22,23,24,25,26]. For example, some existing methods utilize geometric characteristics of the jammed area to localize the jammer [18,19,24,25,27], and these methods require a high density of anchor nodes, typically 100 to 300 nodes in 100 m${}^{2}$. To solve the problem of limited measurements, we take advantage of the jammer’s mobility. As the jammer moves in the NAN, it keeps affecting new smart meters. JamCatcher utilizes the unscented Kalman filter (UKF) and interacting multiple model (IMM) to estimate the state of the jammer as a weighted average of the model’s predicted state and of the new JSS measurements. The main contributions of this paper are presented as follows:

1. As far as we know, JamCatcher is the first work to study the jammer localization problem in an AMI communication network. JamCatcher provides a mobile jammer localization scheme for anti-jamming strategies in the AMI utilizing UAVs.
2. JamCatcher can localize a mobile jammer with a limited number of anchor nodes in a complex environment, while most existed jammer localization methods cannot.
3. Both a real-world experiment and MATLAB simulations are conducted to evaluate JamCatcher. Extensive computer simulations are carried out to compare the localization accuracy of JamCatcher with existing jammer localization methods.

We organize the remainder of the paper as follows. We discuss the related work in Section 2. We introduce related models and formulate the problem in Section 3. In Section 4, we present our framework for localizing a mobile jammer and give the implementation of our algorithms. In Section 5, we validate the proposed localization algorithms through a real-world experiment and extensive MATLAB simulations. Finally, we conclude in Section 6.

## 2. Related Work

Jamming attacks can seriously damage the stability of the smart grid. For instance, a malicious attacker can use jamming attack to damage the accuracy of the smart grid’s state estimation. Deka et al. [28] introduced an undetectable jamming attack that uses measurement jamming in addition to changing meter readings to create a change in state estimation while remaining undetected by the bad-data detection test. Similarly, Guan et al. [7] introduced a false data injection attack that physically changes the system’s state and uses jamming attack to block the wireless transmission channels between sensors and remote estimators, leading to the failure of smart grid’s state estimation. Gai et al. [29] introduced an attack strategy that optimally distributed power usage on both spoofing and jamming attacks to increase the adversarial implication to the wireless communications of the smart grid. Some recent research applied the game theory to study jamming attacks against the smart grid’s state estimation and developed defense strategies in the smart grid [6,30]. A few works have been focused on studying the possibility of manipulating the power market by jamming the pricing signal [4,5,6]. Although jamming attacks in the smart grid have drawn much attention, defense strategies are only given theoretically, and so, an anti-jamming scheme aimed at practical use is an urgent need.

Accurate jammer localization plays an essential role in applying various anti-jamming countermeasures [31]. Based on the localization methodology, the existing jammer localization algorithms can be categorized into range-based methods [16,17,20,21,22,23,26,32] and range-free methods [18,19,24,25,27]. In the range-based methods, the distance between the jammer and the anchor nodes derived from measurements is utilized to estimate the location of the jammer. Range-free methods usually rely on the geometric characteristics of the jammed area to locate the jammer. Prior research has addressed jammer localization problems in different scenarios, such as wireless local area networks (WLAN) [20,21,22], wireless sensor networks (WSN) [23,24,25], and mobile ad-hoc networks (MANET) [26]. Localizing a jammer in an AMI communication network is different from that in other networks due to the large scale and sparse distribution of the anchor nodes. Existing jammer localization methods are not applicable to AMI for three reasons. First, the accuracy of most existing methods relies on a high density of anchor nodes, e.g., range-free methods require typically 100 to 300 nodes in 100 m${}^{2}$ [18,19]. The performance of existing methods degrades evidently as the node density goes extremely low. Second, existing methods assume a sink node far from the jammed area, which can collect JSS measurements from sufficient anchor nodes. However, this is not practical for localizing a mobile jammer in reality since the time delay can be large. Utilizing a tracker to enter the jammed area and collect real-time measurements is a good way to solve the problem. Third, existing methods are developed for localizing stationary jammers, and they consider the JSS measurements or the jammed area time-invariant and obtain the estimated jammer location through static estimations [16,17,18,19,20,21,22,23,24,25,26], which is not applicable to localizing mobile jammers. The most similar to our work, Wu et al. [33] proposed a radio-based robot localization method with low-density WLAN access points (APs). They installed ten APs in an area with a size of 54 m × 53 m, and their positioning system only selected two APs from the detected AP set for localization. However, they used an RSS fingerprinting approach, and their method required 236 anchor nodes to measure the AP signal and build a radio map. Building a radio map for jammer localization is unrealistic since the jammer is non-cooperative and its transmitting power is unknown. As far as we knew, we present here the first work to design a mobile jammer localization framework for anti-jamming technology in the AMI communication network utilizing UAVs. We propose localization algorithms that can localize mobile jammers with complex motion and can achieve good accuracy when the anchor nodes are sparsely distributed.

## 3. Problem Overview

This paper focuses on developing a mobile jammer localization scheme and corresponding localization algorithms for anti-jamming technology in low-density AMI communication networks using mobile trackers. We assume the network is able to identify jamming attacks leveraging the existing jamming detection approaches [34,35]. In this section, we explain the jamming attack scenario in AMI networks and give the assumptions of our work. Then, we formulate the jammer localization problem as a discrete-time state estimation problem.

### 3.1. Localizing the Mobile Jammer in AMI

The AMI communication network features a hierarchical structure (shown in Figure 1), including home area network (HAN), neighbor area network (NAN), and wide area network (WAN). The HAN is composed of smart devices within a home, and a smart meter acts as a gateway in an HAN. The NAN is composed of smart meters, access points, and data collectors installed in the power supply area. The smart meters form a mesh network using wireless communication technologies such as ZigBee and WiFi. Each access point collects data from smart meters in a specific area and delivers them to the data collector. In case the communication link is in a poor condition, each smart meter can act as a repeater and deliver the data in a multi-hop way. Data collectors deliver the data from access points to the control center through the WAN.

Jamming attacks usually occur in the NAN and interfere with the communication between smart meters. The jammer moves within the network and transmits the jamming signal at a constant power level. When the signal-to-noise ratio (SNR) of a wireless link is lower than a threshold, the two nodes will not be able to communicate. According to [36], the nodes under jamming attack can be divided into three types, i.e., jammed node, boundary node, and unaffected node.

- Unaffected node: Unaffected nodes are nodes that can receive packets from all of their neighbors after jamming is present.
- Jammed node: Jammed nodes are nodes that cannot receive messages from any unaffected nodes.
- Boundary node: Boundary nodes are nodes that can receive packets from a part of, but not all of their neighbors.

Jammed nodes and boundary nodes form a jammed area, where nodes can take good measurements of the jamming signal directly. Different from most existing jammer localization algorithms, which only utilize measurements from boundary nodes, JamCatcher makes use of measurements from both boundary nodes and jammed nodes. To be specific, JamCatcher divides the jammer localization process into two stages, i.e., far-field chasing and near-field capturing. The far-field chasing stage starts from the time when the tracker is released in the NAN. In this stage, the tracker is unaffected by the jammer (i.e., out of the jammed area) and can receive JSS measurements from all the boundary nodes through multi-hop transmission. The near-field capturing stage starts as the tracker enters the jammed area. In this stage, the tracker itself becomes an affected node. It will only be able to collect JSS measurements from its neighbors through one-hop communication. The neighbor nodes that the tracker can communicate with include boundary nodes and jammed nodes (when the tracker is close to the jammed node and the SNR is above the threshold).

### 3.2. Related Models

We assume smart meters are able to measure the jamming signal utilizing the method proposed in [17]. Each smart meter is aware of its own position using generalized pattern search (GPS) or some other similar, but less burdensome localization algorithms [37,38]. When the jammer moves in the NAN, affected smart meters can measure the JSS and send the measurements to the tracker. Whether the tracker can receive the messages from smart meters depends on the SNR. In the rest of the paper, observation nodes refer to affected smart meters with which the tracker can communicate. Some related models are given as follows.

The mobile jammer we try to localize has the following characteristics.

Mobile: The jammer moves within a 2D plane with arbitrary speed and towards arbitrary directions. Both the speed and moving directions can not be measured.

Unknown transmit power: The jammer transmits signals at a constant power level and will not change over time. The transmit power level of the jammer is unknown.

Omnidirectional: The jammer is equipped with an omnidirectional antenna.

Based on the characteristics of the AMI network, we examine the smart meters with the following characteristics.

Multi-hop: The smart meters communicate with each other in a multi-hop fashion and form a large-scale wireless mesh network.

Location-aware: Each smart meter is aware of its own location.

Measurement capability: Each smart meter can measure the JSS using methods proposed in [17].

To describe the wireless channel characteristics, we use the log-normal shadowing model, which captures both path loss and shadowing. Let $P_{0}$ be the reference power of the source at reference distance $d_{0}$. Then, the received signal power $P_{r}$ at the anchor node in dBm at a distance of *d* can be given as:

(1) $$P_{r}=P_{0}-10\eta \log_{10}\left(\frac{d}{d_{0}}\right)+X_{\sigma }$$

where $X_{\sigma }$, caused by shadowing, is a Gaussian zero-mean random variable with standard deviation σ, and η is the path loss exponent and can be obtained through empirical measurements. $P_{0}$ depends on the transmit power of the source, and $d_{0}$ is typically 1 m. In a free space, η is two, and $X_{\sigma }$ is always zero.

### 3.3. Problem Formulation

The jammer’s motion can be described as a non-linear discrete-time controlled process that is governed by the stochastic differential equation:

(2) $$x_{t}=f\left(x_{t-1}\right)+w_{t}$$

where $w_{t}$ is the model noise with a zero mean Gaussian distribution, i.e., $w_{t}∼N\left(0,Q\right)$. f(·) is the transition function that describes the jammer’s state change. For example, if the jammer is moving with constant velocity and we use $x_{t}={\left[x_{t},y_{t},v_{xt},v_{yt}\right]}^{T}$ to describe the jammer’s state at time *t*, in which $x_{t}$ and $y_{t}$ represent the position coordinates of the jammer and $v_{xt}$ and $v_{yt}$ represent the linear velocity of the jammer, then the motion model will be:

(3) $$x_{t}=\left(\begin{array}{cccc} 1 & 0 & T & 0\\ 0 & 1 & 0 & T\\ 0 & 0 & 1 & 0\\ 0 & 0 & 0 & 1 \end{array}\right)x_{t-1}+w_{t}$$

where $x_{t}={\left[x_{t},y_{t},v_{xt},v_{yt}\right]}^{T}$ and the *T* is the sampling time interval.

The state of the jammer cannot be directly measured, but we can use some sensors to give observations of the jammer. The observation $z_{t}$ can be described as:

(4) $$z_{t}=h\left(x_{t}\right)+v_{t}$$

where random variable $v_{t}$ is the measurement noise with a zero mean Gaussian distribution, i.e., $v_{t}∼N\left(0,R\right)$. h(·) is the observation function, as we assume the smart meters can measure the jamming signal strength of the jammer, and it can be described using Equation (1). For example, assume that at time *t*, a jammer located at $\left(x_{J_{t}},y_{J_{t}}\right)$ starts to transmit at the power level of $P_{J}$ and that *m* nodes located at ${\left\{\left(x_{i},y_{i}\right)\right\}}_{i\in [1,m]}$ can measure the JSS. Then, $z_{t}=\left[z_{t}^{1},z_{t}^{2},\ldots ,z_{t}^{m}\right]$, where $z_{t}^{i}$ is the observation given by node *i* and can be given as:

(5) $$\begin{array}{c} \begin{array}{cc} z_{t}^{i} & =P_{J}-10\eta \log_{10}\left(d_{i}\right)+v_{t}\\ d_{i} & =\sqrt{{\left(x_{J_{t}}-x_{i}\right)}^{2}+{\left(y_{J_{t}}-y_{i}\right)}^{2}} \end{array} \end{array}$$

Then, the jammer localization problem can be described as estimating the jammer’s position $\left(x_{J_{t}},y_{J_{t}}\right)$ at time *t* given the observation sequence $z_{1:t}$, where $z_{1:t}$ represents the observations from Time 1 to time *t*.

## 4. Design

JamCatcher divides the jammer localization process into two stages, i.e., far-field chasing stage and near-field capturing state. The far-field chasing stage is just like the situations in existing research, where measurements can be obtained from sufficient observation nodes. However, the time delay is large in this stage. JamCatcher uses the genetic algorithm for finding the best estimation of the jammer’s location. Instead of localizing the jammer accurately, far-field stage algorithms aim at driving the tracker as close as possible to the jammer in a short time. In the near-field stage, the tracker can take measurements from limited observation nodes in real time. JamCatcher provides a dynamic algorithm and a static algorithm for the near-field capturing stage. The dynamic algorithm takes advantages of the jammer’s mobility and gives estimate of the jammer’s location based on a dynamic model that describes the jammer’s motion and measurements from different time. The static algorithm is a least squares-based method that uses measurements from the current time. It provides a raw estimate of the jammer’s state for the dynamic algorithm. An unscented Kalman filter-based data fusion algorithm is used to alleviate the impact of noise and give a refined estimate based on the outputs of the static algorithm and dynamic algorithm.

### 4.1. Workflow of JamCatcher

**Figure 2:**
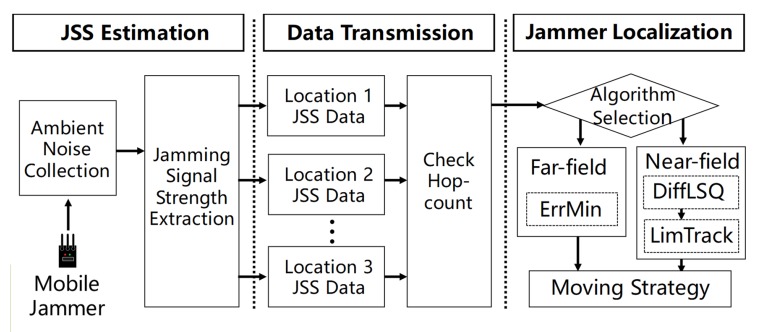
Workflow of JamCatcher. JSS, jamming signal strength; ErrMin, error minimizing.

JamCatcher is a localization scheme for the tracker to cope with the AMI network nodes to localize a mobile jammer in real time. As shown in Figure 2, the workflow of JamCatcher can be roughly divided into three steps:

- JSS estimation: As the jammer moves in the AMI network, smart meters within the jammed area become affected nodes. Every smart meter can obtain its state by checking whether it has lost some of its neighbors; if yes, it will label itself as an affected node. Each affected node measures JSS periodically and stores it with a timestamp. Similar to received signal strength (RSS), JSS reflects the distance between the jammer and the affected node. JSS cannot be measured directly since jamming signals are mixed with signals transmitted by regular wireless devices. However, it can be estimated as the average of the ambient noise floor (ANF) [17], which is the measurement of the ambient noise. The tracker is also capable of measuring JSS; thus, the tracker itself can act as an observation node with mobility.
- Data transmission: When the tracker wants to collect JSS measurements, it broadcasts a request. On receiving the request message, each affected node sends a response to the tracker. The response message includes JSS measurement, node location, hop-count, and a timestamp. After receiving the response messages from observation nodes, the tracker judges which state it is in by checking the hop-counts from the messages. Specifically, the tracker is in the far-field chasing stage if most hop-counts are larger than one, which means the tracker has not entered the jammed area. The tracker is in the near-field capturing stage if most hop-counts are one, which means it has entered the jammed area.
- Jammer localization: The tracker chooses among the localization algorithms according to the stage i which it is. In the far-field chasing stage, the tracker is far away from the jammer and is surrounded by unaffected nodes; thus, it can obtain JSS measurements from all the smart meters on the boundary of the jammed area. However, the delay of the measurements is large due to multi-hop transmission. Instead of localizing the jammer accurately, far-field stage algorithms aim at driving the tracker as close as possible to the jammer in a short time. In the near-field capturing stage, the tracker has entered the jammed area and can collect measurements from affected nodes directly, but the number of nodes it can communicate with is limited. The near-field capturing stage can be further divided into two situations, depending on the number of nodes with which the tracker can communicate.

As the algorithm in the far-field chasing stage is not the focus of this work, we utilized the error-minimizing jammer localization method propose by [17] (ErrMin) and improved their method in two aspects. First, we developed an evaluation metric that does not contain transmit power as a parameter and thus reduces the searching space prominently. Secondly, we combined the developed evaluation metric with the genetic algorithm (GA) and proposed an effective jammer localization mechanism. ErrMin is a non-real-time jammer localization algorithm for the tracker to move into the jammed area as soon as possible.

The algorithms in the near-field capturing stage cope with the situation when the tracker enters the jammed area and can only communicate with limited observation nodes. A static algorithm (named DiffLSQ), as well as a dynamic algorithm (named LimTrack) were developed for localizing the jammer in this stage. When the tracker can collect JSS from more than two observation nodes, the static algorithm combined with the dynamic algorithm were used for localization, and the output of the static algorithm was taken as the input of the dynamic algorithm. When the tracker can collect measurements from less than two nodes, only the dynamic algorithm was used. The details of the algorithms are described in the following subsections.

### 4.2. Far-Field Chasing Algorithm

The far-field chasing algorithm works as follows. For a given set of JSS measurements, it searches through the target area and evaluates each possible location. Based on the JSS measurements, the algorithm gives every candidate location a quantitative evaluation, which indicates how close it is to the true jammer location. Assume a jammer located at $\left(x_{J},y_{J}\right)$ starts to transmit at the power level of $P_{J}$ and *m* nodes located at ${\left\{\left(x_{i},y_{i}\right)\right\}}_{i\in [1,m]}$ become observation nodes. For a given location $\left({\hat{x}}_{J},{\hat{y}}_{J}\right)$, each observation node can estimate the jammer’s transmit power according to Equation (1):

(6) $$\begin{array}{c} \begin{array}{cc} {\hat{P}}_{J_{i}} & =P_{r_{i}}+{\hat{P}}_{L_{i}}\left({\hat{d}}_{i}\right)\\ {\hat{P}}_{L_{i}}\left({\hat{d}}_{i}\right) & =10\eta \log_{10}\left({\hat{d}}_{i}\right)\\ {\hat{d}}_{i} & =\sqrt{{\left({\hat{x}}_{J}-x_{i}\right)}^{2}+{\left({\hat{y}}_{J}-y_{i}\right)}^{2}} \end{array} \end{array}$$

**Figure 3:**
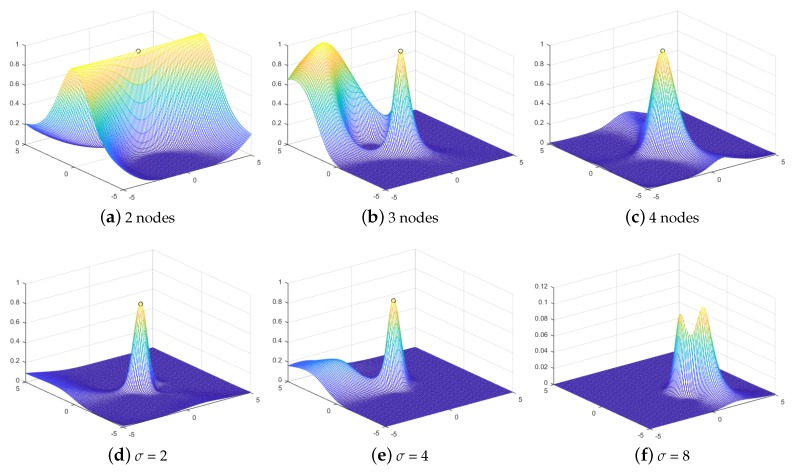
The value of the metric on different locations within the rectangular grid of [−5,5]×[−5,5].

For a set of boundary nodes, we can get a set of estimated jammer transmit powers $\left\{{\hat{P}}_{J_{1}},{\hat{P}}_{J_{2}},\ldots ,{\hat{P}}_{J_{m}}\right\}$. If the chosen location is exactly the jammer’s true location and the noise is sufficiently small, we will have ${\hat{P}}_{J_{1}}={\hat{P}}_{J_{2}}=\ldots ={\hat{P}}_{J_{m}}$. When the chosen location is away from the true location, the variance of ${\left\{{\hat{P}}_{J_{i}}\right\}}_{i\in [1,m]}$ becomes larger than zero. Thus, we use the standard deviation as the evaluation metric and map it to the interval of [0,1].

(7) $$e_{s}=exp\left\{-\sqrt{\frac{1}{m}\sum_{i=1}^{m}{\left({\hat{P}}_{J_{i}}-{\hat{\bar{P}}}_{J_{i}}\right)}^{2}}\right\}$$

where ${\hat{\bar{P}}}_{J_{i}}$ is the mean of ${\left\{{\hat{P}}_{J_{i}}\right\}}_{i\in [1,m]}$. Figure 3 shows the value of the metric at different locations within the rectangular grid of [−5,5]×[−5,5], where eight observation nodes are located over the rectangular grid of [−3.75,3.75]×[−1.25,1.25] with a grid size of 2.5 and the jammer located at (0,0). We observed that when the number of observation nodes decreased to under four, there was a high possibility that ErrMin failed to give the correct estimate since a global minimum did not exist. The performance of ErrMin degraded as the measurement noise increased.

After developing the evaluation metric, finding the best estimation is equal to finding a location that has the highest score on the metric. As a matter of fact, the jammer will always locate inside the group of affected nodes since the jammer we studied was omnidirectional. Thus, the searching space can be reduced to the circle formed by the affected nodes. We adopted GA to search for this location automatically. We mapped the locations in the jammer area into genetic representations and defined the fitness function as Equation (7). After obtaining the estimated position of the jammer, we can estimate the jammer’s transmit power $P_{J}$ as the mean of ${\left\{{\hat{P}}_{J_{i}}\right\}}_{i\in [1,m]}$, i.e., ${\hat{P}}_{J}=\frac{1}{m}\sum_{i=1}^{m}{\hat{P}}_{J_{i}}$.

### 4.3. Near-Field Capturing Algorithms

We developed a static algorithm and a dynamic algorithm for the near-field capturing process. The static algorithm only takes current JSS measurements as the input and estimates the jammer’s location. The dynamic algorithm includes a dynamic model that describes the jammer’s motion and an unscented Kalman filter (UKF)-based data fusion algorithm. It starts from an initial estimated jammer state, which is set manually, and gives an estimate using the motion model at the beginning of each time step. Then, it uses JSS measurements of the current time to revise the estimate. Since the estimated jammer location is a weighted average of the predicted location using the motion model and the new JSS measurements, it deals effectively with the uncertainty due to noisy sensor data. However, the dynamic algorithm is sensitive to the initial given state due to the strong nonlinearity of the observation function (see Equation (1)) in our jammer localization scenario. To solve this problem, we used the static algorithm to generate the initial state of the dynamic algorithm. Furthermore, we combined the two algorithms to obtain a refined estimation of the jammer’s location when the number of observation nodes was more than two.

**Figure 4:**
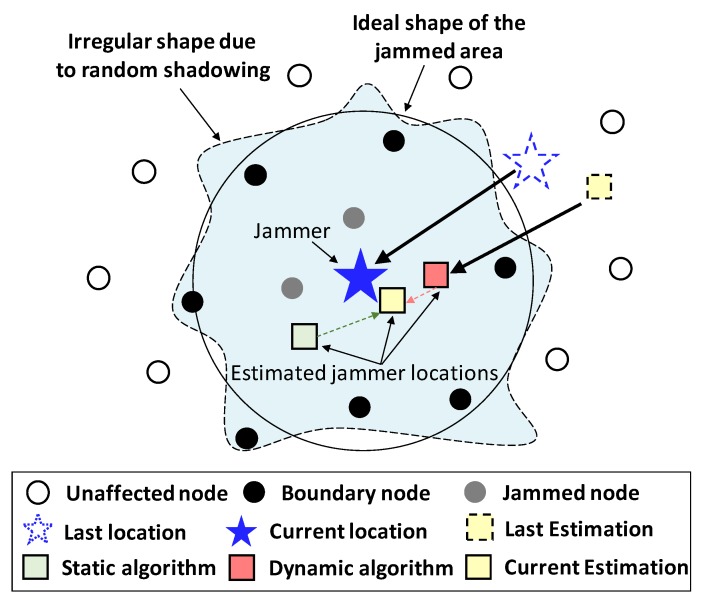
Illustration of near-field capturing algorithms.

Figure 4 shows the workflow of the near-field algorithm. At each time step, the dynamic algorithm gives an estimation based on the last estimated jammer location and the dynamic model. At the same time, the static algorithm gives an estimation based on current JSS measurements. Then, the output of static algorithm, along with the current JSS measurements are used as the inputs of the UKF, and the output is the current estimation of the jammer’s location. When the observation nodes are less than two, only the dynamic algorithm is utilized to localize the jammer.

#### 4.3.1. Static Algorithm (DiffLSQ)

We propose a least-squares method DiffLSQ to localize the jammer when the tracker can collect JSS from more than two affected nodes. Then, based on the log-normal shadowing model, the measured JSS at affected node *i* located at $\left(x_{i},y_{i}\right)$ can be written as:

(8) $$P_{r_{i}}=P_{J}-10\eta \log_{10}\left(d_{i}\right)+X_{\sigma }$$

where $d_{i}$ is the distance between affected node *i* and the jammer. As the transmit power of jammer $P_{J}$ can be estimated in the far-field chasing stage using ErrMin, $d_{i}$ can be estimated as:

(9) $${\hat{d}}_{i}=10^{\frac{{\hat{P}}_{J}-P_{r_{i}}}{10\eta }}$$

Therefore, we can obtain the following formula,

(10) $${\left({\hat{x}}_{J}-x_{i}\right)}^{2}+{\left({\hat{y}}_{J}-y_{i}\right)}^{2}={\hat{d}}_{i}^{2}$$

where $\left(x_{i},y_{i}\right)$ is the position of affected node *i* and $\left({\hat{x}}_{J},{\hat{y}}_{J}\right)$ is the position of the jammer.

In the above equation, the unknown variables include ${\hat{x}}_{J}$ and ${\hat{y}}_{J}$. Suppose at time *t* that the tracker can collect JSS from *n* nodes; we can have *n* equations:

(11) $$\begin{array}{cc} {\left(x_{1}-{\hat{x}}_{J_{t}}\right)}^{2}+{\left(y_{1}-{\hat{y}}_{J_{t}}\right)}^{2} & ={\hat{d}}_{1,t}^{2}\\ {\left(x_{2}-{\hat{x}}_{J_{t}}\right)}^{2}+{\left(y_{2}-{\hat{y}}_{J_{t}}\right)}^{2} & ={\hat{d}}_{2,t}^{2}\\ & \vdots \\ {\left(x_{n}-{\hat{x}}_{J_{t}}\right)}^{2}+{\left(y_{n}-{\hat{y}}_{J_{t}}\right)}^{2} & ={\hat{d}}_{n,t}^{2} \end{array}$$

To avoid solving complicated nonlinear equations, we first linearized the problem by subtracting the $n^{\mathrm{th}}$ equation from both sides of the first n−1 equations and obtained linear equations:

(12) $$\begin{array}{cc} \left(\;x_{1}^{2}\;-\;x_{n}^{2}\;\right) & -2\left(\;x_{1}\;-\;x_{n}\;\right){\hat{x}}_{J_{t}}\;+\;\left(\;y_{1}^{2}-y_{n}^{2}\;\right)\;-\;2\left(\;y_{1}-y_{n}\;\right){\hat{y}}_{J_{t}}\\ & ={\hat{d}}_{1,t}^{2}-{\hat{d}}_{n,t}^{2}\\ \left(\;x_{2}^{2}-x_{n}^{2}\;\right) & \;-\;2\left(\;x_{2}-x_{n}\;\right){\hat{x}}_{J_{t}}\;+\;\left(\;y_{2}^{2}\;-\;y_{n}^{2}\;\right)\;-\;2\left(\;y_{2}-y_{n}\;\right){\hat{y}}_{J_{t}}\\ & ={\hat{d}}_{2,t}^{2}\;-\;{\hat{d}}_{n,t}^{2}\\ & \vdots \\ \left(\;x_{n\;-\;1}^{2}\;-\;x_{n}^{2}\;\right) & \;-\;2\left(\;x_{n\;-\;1}\;-\;x_{n}\;\right){\hat{x}}_{J_{t}}\;\;\;+\;\left(\;y_{n\;-\;1}^{2}\;-\;y_{n}^{2}\;\right)\;-\;2\left(\;y_{n\;-\;1}\;-\;y_{n}\;\right){\hat{y}}_{J_{t}}\\ & ={\hat{d}}_{n\;-\;1,t}^{2}\;-\;{\hat{d}}_{n,t}^{2} \end{array}$$

Then, this can be written in the form of Ax=b with:

$$\begin{array}{c} A\;=\;\left(\begin{array}{cc} x_{1}-x_{n} & y_{1}-y_{n}\\ \vdots & \vdots \\ x_{n-1}-x_{n} & \;y_{n-1}-y_{n} \end{array}\right) \end{array}$$

and:

$$\begin{array}{c} b\;=\;\frac{1}{2}\left(\begin{array}{c} \left(x_{1}^{2}\;-\;x_{n}^{2}\right)+\left(y_{1}^{2}\;-\;y_{n}^{2}\right)\;-\;\left({\hat{d}}_{1,t}^{2}\;-\;{\hat{d}}_{n,t}^{2}\right)\\ \vdots \\ \left(x_{n\;-\;1}^{2}\;-\;x_{n}^{2}\right)\;+\;\left(y_{n\;-\;1}^{2}\;-\;y_{n}^{2}\right)\;-\;\left({\hat{d}}_{n\;-\;1,t}^{2}\;-\;{\hat{d}}_{n,t}^{2}\right) \end{array}\right) \end{array}$$

We can estimate the location of the jammer at time *t* using the least squares (LSQ) method,

(13) $$x={\left[{\hat{x}}_{J_{t_{m}}},{\hat{y}}_{J_{t_{m}}}\right]}^{T}={\left(A^{T}A\right)}^{-1}A^{T}b$$

From the above, we can infer that in order to solve the problem, we need at least three equations. Because the tracker itself is also able to estimate JSS through ambient noise measurement, the tracker needs to collect JSS from at least two affected nodes.

#### 4.3.2. Dynamic Algorithm (LimTrack)

The dynamic algorithm contains two parts. The first part is a motion model, which describes the jammer’s motion. The second part is a UKF based data fusion algorithm that produces an estimate of the state of the jammer as an average of the model’s predicted state and of the new measurements using a weighted average.

The jammer’s motion is usually complicated in AMI networks. For example, the attacker carrying a jammer on his/her car may change the driving direction at any crossing on the street. Thus, it is not applicable to simply treat the jammer’s speed as a constant variable. To model the jammer’s motion, we utilized the interacting multiple model (IMM) [39]. The IMM algorithm selects multiple parallel running models and then automatically switches between the models according to the Markov transition probability matrix. As a matter of fact, the jammer’s motion is usually bounded by the road it moves along, which means the jammer is either in a linear motion (when the road is straight) or in a turning motion (when there is a turn). Thus, we used a constant velocity (CV) model and a coordinate turn (CT) model to describe the jammer’s motion. The CV model is defined as:

(14) $$x_{t}=\left(\begin{array}{cccc} 1 & 0 & T & 0\\ 0 & 1 & 0 & T\\ 0 & 0 & 1 & 0\\ 0 & 0 & 0 & 1 \end{array}\right)x_{t-1}+w_{t}$$

where $x_{t}={\left[x_{t},y_{t},v_{xt},v_{yt}\right]}^{T}$ and the *T* is the sampling time interval. The CT model is:

(15) $$x_{t}=\left(\begin{array}{cccc} 1 & 0 & \frac{\sin(\omega T)}{\omega } & \frac{\cos(\omega T)\;-\;1}{\omega }\\ 0 & 1 & \frac{1\;-\;\cos(\omega T)}{\omega } & \frac{\sin(\omega T)}{\omega }\\ 0 & 0 & \cos(\omega T) & \sin(\omega T)\\ 0 & 0 & -\;\sin(\omega T) & \cos(\omega T) \end{array}\right)x_{t\;-\;1}\;+\;w_{t}$$

where ω is the angular velocity (rad/s).

**Figure 5:**
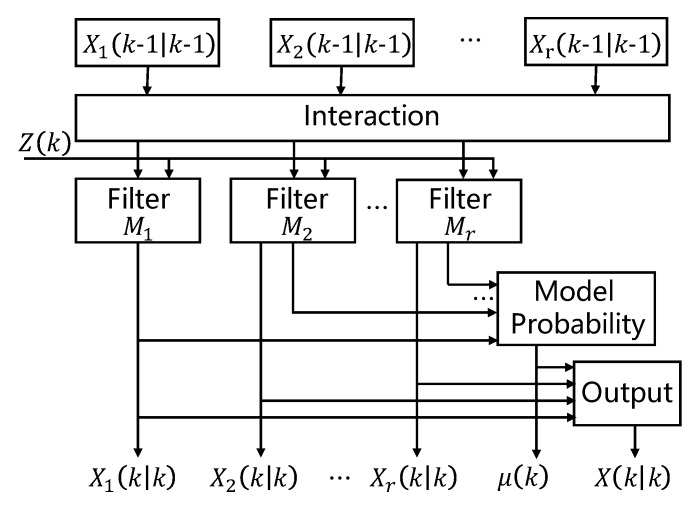
Framework of the interacting multiple model (IMM) algorithm.

Figure 5 shows the framework of the IMM algorithm. In the input state, elemental filters (models) interact with one another by utilizing probabilistically-weighted sums of the most recent estimates from all elemental filters as their inputs. Then, the weights of elemental filters are updated based on the outputs of each filter. The output is also the probabilistically-weighted sums of the outputs from all elemental filters.

The UKF addresses the general problem of estimating the state x of a non-linear discrete-time controlled process that is governed by the stochastic differential equation given in Equation (2) with an observation z given in Equation (4). The motion of the jammer is described using the IMM. The initial estimated state of the jammer is obtained from the static algorithm. The observation function in our algorithm contains two parts, i.e., $Z_{k}={\left[z_{t}^{1},z_{t}^{2}\right]}^{T}$, where $z_{t}^{1}={\left[{\hat{x}}_{t},{\hat{y}}_{t}\right]}^{T}$ is the output of the static algorithm. As the state of the jammer is $x_{t}={\left[x_{t},y_{t},v_{xt},v_{yt}\right]}^{T}$, $z_{t}^{1}$ can be written as:

(16) $$z_{t}^{1}=\left(\begin{array}{cccc} 1 & 0 & 0 & 0\\ 0 & 1 & 0 & 0\\ 0 & 0 & 0 & 0\\ 0 & 0 & 0 & 0 \end{array}\right)x_{t}$$

The observation function $z_{t}^{2}$ is a set of JSS measurements from *m* observation nodes, i.e.,

(17) $$z_{t}^{2}={\left[P_{r_{1}}\left(x_{t}\right),P_{r_{2}}\left(x_{t}\right),\ldots ,P_{r_{m}}\left(x_{t}\right)\right]}^{T}$$

For an observation node located at $\left(x_{oi},y_{oi}\right)$, $P_{r_{i}}\left(x_{t}\right)$ is defined as:

(18) $$P_{r_{i}}\left(x_{t}\right)=P_{J}-10\eta \log_{10}\left(\sqrt{{\left(x_{t}-x_{oi}\right)}^{2}+{\left(y_{t}-y_{oi}\right)}^{2}}\right)$$

The UKF uses a deterministic sampling technique called unscented transform to select a minimal set of sample points (i.e., sigma points) around the mean. Each sigma point represents an estimation of the true state and is propagated through the non-linear functions. Then, the new estimation is given as the weighted mean of the sigma points. The details are given as follows:

1. State initiation:
  (19) $$\begin{array}{cc} {\hat{x}}_{0} & =E[x_{0}]\\ P_{0} & =E[\left(x_{0}-{\hat{x}}_{0}\right){\left(x_{0}-{\hat{x}}_{0}\right)}^{T}] \end{array}$$
  where ${\hat{x}}_{0}$ is the initial estimated jammer state (given by ErrMin) and $P_{0}$ is the initial error covariance matrix.
2. Sigma points’ calculation:
  (20) $$\left\{\begin{array}{cc} X_{0,t\;-\;1}\;=\;{\hat{x}}_{t\;-\;1}, & \;\omega_{0}\;=\;\lambda /\left(\;L\;+\;\lambda \;\right)\\ X_{i,t\;-\;1}\;=\;{\hat{x}}_{t\;-\;1}\;+\;{\left(\sqrt{\;\left(\;L+\lambda \;\right)P_{t\;-\;1}}\;\right)}_{i}, & \;\omega_{i}\;=\;0.5/\left(\;L\;+\;\lambda \;\right)\\ X_{i\;+\;L,t\;-\;1}\;=\;{\hat{x}}_{t\;-\;1}\;-\;{\left(\;\sqrt{\;\left(\;L+\lambda \;\right)P_{t\;-\;1}}\;\right)}_{i}, & \;\omega_{i\;+\;L}\;=\;0.5/\left(\;L\;+\;\lambda \;\right) \end{array}\right.$$
  where i=1,2,…,L, *L* is the dimension of state x, λ is a parameter used to adjust ω, and $P_{t}$ is the a posteriori error covariance matrix at time t−1.
3. State update:
  (21) $$\begin{array}{c} X_{i,t|t\;-\;1}\;=\;f\left(X_{i,t\;-\;1}\right)\\ {\hat{x}}_{t|t\;-\;1}\;=\;\sum_{i=0}^{2L}\;\omega_{i}X_{i,t|t\;-\;1}\\ P_{t|t\;-\;1}\;=\;\sum_{i=0}^{2L}\;\omega_{i}\left[X_{i,t|t\;-\;1}\;-\;{\hat{x}}_{t|t\;-\;1}\right]{\left[X_{i,t|t\;-\;1}\;-\;{\hat{x}}_{t|t\;-\;1}\right]}^{T}\;\;+\;Q_{t}\\ Y\;=\;h(X_{i,t|t\;-\;1})\\ {\hat{y}}_{t|t\;-\;1}\;=\;\sum_{i=0}^{2L}\;\omega_{i}Y_{i,t|t\;-\;1} \end{array}$$
4. Measurement update:
  (22) $$\begin{array}{c} P_{yy}\;=\;\sum_{i=0}^{2L}\;\omega_{i}\left[Y_{i,t|t\;-\;1}\;-\;{\hat{y}}_{t|t\;-\;1}\right]{\left[Y_{i,t|t\;-\;1}\;-\;{\hat{y}}_{t|t\;-\;1}\right]}^{T}\;+\;R_{t}\\ P_{xy}\;=\;\sum_{i=0}^{2L}\;\omega_{i}\left[X_{i,t|t\;-\;1}\;-\;{\hat{x}}_{t|t\;-\;1}\right]{\left[Y_{i,t|t\;-\;1}\;-\;{\hat{y}}_{t|t\;-\;1}\right]}^{T}\\ K_{t}=P_{xy}P_{yy}^{-1}\\ {\hat{x}}_{t}={\hat{x}}_{t|t-1}+K_{t}\left(y_{t}-{\hat{y}}_{t|t-1}\right)\\ P_{t}=P_{t|t\;-\;1}-K_{t}P_{yy}K_{t}^{T} \end{array}$$

The output of UKF is an a posteriori state estimate ${\hat{x}}_{t}$ of the jammer and an a posteriori error covariance matrix $P_{t}$, which is a measure of the estimated accuracy of the state estimate.

## 5. Evaluation

We evaluated JamCatcher by conducting both a real-world experiment and MATLAB simulations. The data collected from a real-world experiment were used for evaluating both the far-field chasing algorithm and near-field capturing algorithm. To evaluate JamCatcher in a larger scale scenario, we conducted extensive MATLAB simulations. As the far-field algorithm is not the focus of this paper, we only evaluated near-field algorithms in the simulations. The performance of JamCatcher was compared to some existing jammer localization algorithms. However, most existing algorithms cannot localize the jammer with limited observation nodes (e.g., less than four). Thus, we chose adaptive LSQ (or ALSQ) [16] and ErrMin [17] for comparison, as they require less observation nodes compared to other existing methods. Note that ALSQ and ErrMin are not applicable to the near-field capturing stage where observation nodes could be less than two, so we relaxed the conditions and allowed them to take measurements from more observation nodes compared to JamCatcher (details are given in the following experiments). The results of ALSQ and ErrMin were only used as a reference.

### 5.1. Real-World Experiments

#### 5.1.1. Data Collection

**Figure 6:**
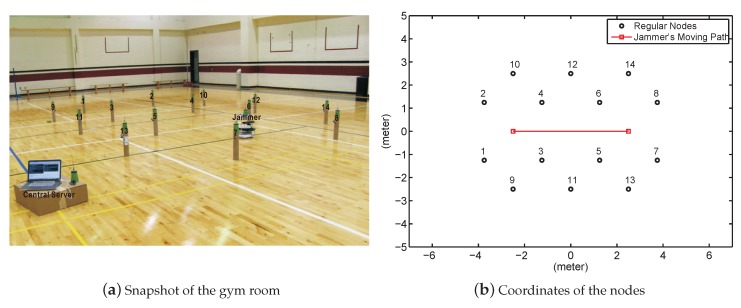
Network deployment in an indoor gym room.

We conducted all experiments in a 20-by-30 m gymnasium. Our experiment involved 14 nodes and a mobile jammer, which were implemented on MicaZ nodes. The deployment layout is illustrated in Figure 6; each node measured the jamming signal strength (JSS) once every second and stored the measured JSS and its timestamp in a local memory. A central server was implemented on a laptop. Connected with a sink node, the server collected the data from all nodes. We mounted one constant jammer on a robot that moved back and forth on a line between [−2.5,0] and [2.5,0]. In particular, the constant jammer traveled from [−2.5,0] to [2.5,0] 11 times and from [2.5,0] to [−2.5,0] 11 times. In total, we collected JSS when the jammer was at many different locations in the 22 independent experiments. Additionally, we recorded the true location of the jammer in real time by setting up a surveillance video camera overlooking the entire network field. Given the collected JSS measurements and their timestamps, we estimated the jammer’s locations utilizing our proposed localization algorithms.

The collected data were used for both the far-field chasing algorithm and the near-field capturing algorithm. Instead of making a real tracker, we assumed there was a virtual tracker that could collect data from the 14 nodes and localize the jammer using the JSS measurements. For far-field chasing algorithm, all the data collected from 14 nodes were used. We compared the performance of far-field chasing algorithm to ALSQ and the original ErrMin proposed in [17], which used the simulated annealing (SA) algorithm to estimate the jammer’s location. For the near-field capturing algorithm, we manually chose observation nodes from the 14 nodes at different times to create situations when there were less than four nodes. Noting that existing methods failed to estimate the jammer’s location when the number of measurements was less than for, for comparison, we fixed the number of observation nodes to eight for the other methods and six at most for JamCatcher.

To evaluate the accuracy of our localization process, we show the Euclidean distance between the estimated jammer’s location and the true jammer’s location. We also used the root mean squared error (RMSE) to analyze the average localization error. We present the cumulation distribution functions (CDF) of the localization error to show the statistical characteristics.

#### 5.1.2. Performance Evaluation

**Figure 7:**
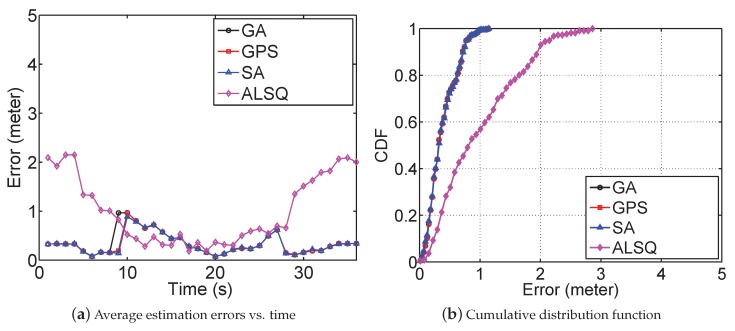
Performance of the far-field chasing algorithm. GPS, generalized pattern search.

The performance of our error-minimizing-based far-field chasing algorithm is shown in Figure 7, which is compared to the performance of ALSQ and the original ErrMin (referred to as SA in Figure 7). For our far-field chasing algorithm, we tested two different searching algorithms, i.e., GA and generalized pattern search (GPS). Figure 7a shows localization errors as the constant jammer moved in a straight line between [−2.5,0] and [2.5,0]. All error-minimizing framework-based algorithms achieved estimation errors less than one meter all the time, while the estimation errors of ALSQ were more than 2 m when the mobile jammer was close to [−2.5,0] and [2.5,0]. Figure 7b depicts the cumulative distribution function of the estimation errors. We observed that the error-minimizing-based method achieved a similar accuracy, and all of them constantly outperformed the ALSQ method in terms of RMSE (from 0.65 m to 0.20 m).

The path loss exponent η is a parameter only related to the environment and can be obtained from empirical measurements. When the value of η used in our algorithm is set to be different from the true value obtained through experiments (i.e., η=2.11), the estimated jammer location may deviate from the true location. To evaluate the impact of errors in the path loss exponent, we set η to different values from 2.0 to 3.0, and the result is shown in Table 1. When η was set to be 3.00, which is the most distant value from the true value, the maximum estimation error (RMSE of 0.31 m) occurred. However, compared to the reference estimation error (RMSE of 0.20 m when η was set to 2.11), it can be seen that the deviation in η had a minor influence on the accuracy of our far-field chasing algorithm.

The performance of our near-field capturing algorithm was compared to the original ErrMin [17]. For ErrMin, we used JSS measurements from eight observation nodes, located over the rectangular grid of [−3.75,3.75]×[−1.25,1.25] with a grid size of 2.5 m. For JamCatcher, six nodes located on the rectangular grid of [−2.5,2.5]×[−1.25,1.25] were used at the beginning, and after every five seconds, one node was removed from the observation node set manually. The initial state of the near-field algorithm of JamCatcher was obtained using the far-field algorithm. Results show that the RMSE of JamCatcher was 0.27 m, which outperformed ErrMin with an RMSE of 0.31 m, even though the average number of observation nodes of JamCatcher was only half as much as ErrMin.

**Figure 8:**
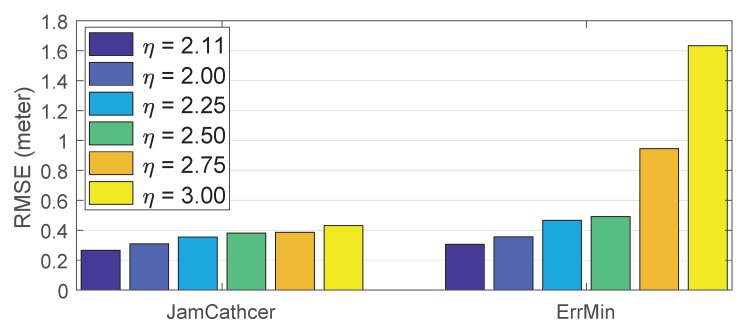
Impact of estimation error in η on the performance of JamCatcher and ErrMin.

To evaluate the influence of the path loss component η on the accuracy of the near-field algorithm, we also changed it from 2.00 to 3.00 and calculated the RMSEs of the estimation errors. Figure 8 shows the results. We can see that the value of the path loss component had a minor influence on our near-field algorithm, which always outperformed the ErrMin algorithm. ErrMin performed similarly when η was set to be under 2.50 (the true value was 2.11). However, when η was set to be larger than 2.50, we found a dramatic increase in the RMSEs (from an average of 0.4 m when η was under 2.50 to an average of 1.3 m). The reason is that ErrMin used searching algorithms to find the location with the highest value on the evaluation metric as the estimated jammer location. As the observation nodes were of limited number and η deviated from the true value, there was a high possibility that other locations had a higher value on the evaluation metric than the true jammer location. In the case of JamCatcher, the impact of errors in η was alleviated using the dynamic model of the jammer’s motion.

### 5.2. MATLAB Simulations

To evaluate JamCatcher in a a larger scale scenario, we conducted extensive MATLAB simulations. In the simulations, we focused on the accuracy of the near-field algorithm of JamCatcher, which was the main contribution of this paper, and compared the performance of JamCatcher to ALSQ and ErrMin. For existing methods to work, we assumed a system adopting ALSQ or ErrMin can obtain JSS measurements from all the boundary nodes and ignore the time delay caused by multi-hop transmission. As for JamCatcher, we set the maximum communication of the tracker to be 60 m. The tracker only collected JSS measurements from nodes within its communication range instead of using JSS measurements from all the boundary nodes. Thus, the number of observation nodes for JamCatcher was always smaller than that for ALSQ and ErrMin at any time step.

#### 5.2.1. Experimental Setup

The parameters of the simulation are shown in Table 2. We simulated a jammer localization process in a 500-by-500 m square AMI network, and the distance between two neighbor smart meters was 40 m. For simplicity, the smart meters were set on the grid points, e.g., (0,0), (40,0), and (40,40), etc. Note that the node density in our simulation (144 nodes in a 500-by-500 m squared area) was much smaller than that in ALSQ [16] or ErrMin [17] (200 nodes in a 300-by-300 m squared area). The path loss exponent η of the shadowing model was set as 2.40, and the standard deviation of the random attenuation σ was set to be 2.0 in order to simulate a highly-irregular radio environment. We set the power of ambient noise as −65 dBm, and when the measured JSS on a smart meter was less than −65 dBm, we considered the smart meter an unaffected node. Thus, the jamming range of the jammer was around 80 m. We set an SNR threshold of −3 dBm; when the SNR was under the threshold, the communicating parties would not be able to receive messages from each other.

The simulation process was done in following steps. The jammer started from the initial location (150,0) and moved at a linear velocity of 5 m/s and a angular velocity of 0.0314 rad/s. The tracker started from (100,0) and kept moving towards the jammer with a linear speed of 6 m/s. At each time step, the mobile jammer created a jammed area within which affected nodes (including jammed nodes and boundary nodes) were located. All the boundary nodes were chosen as observation nodes for ALSQ and ErrMin. As for JamCatcher, we calculated the SNR for each affected node located within the tracker’s communication range. If the SNR was larger than the threshold, the affected node was considered an observation node. Each simulation lasted for 200 time steps and was repeated 20 times, then we calculated the RMSEs of each algorithm.

#### 5.2.2. Performance Comparison and Results Analysis

**Figure 9:**
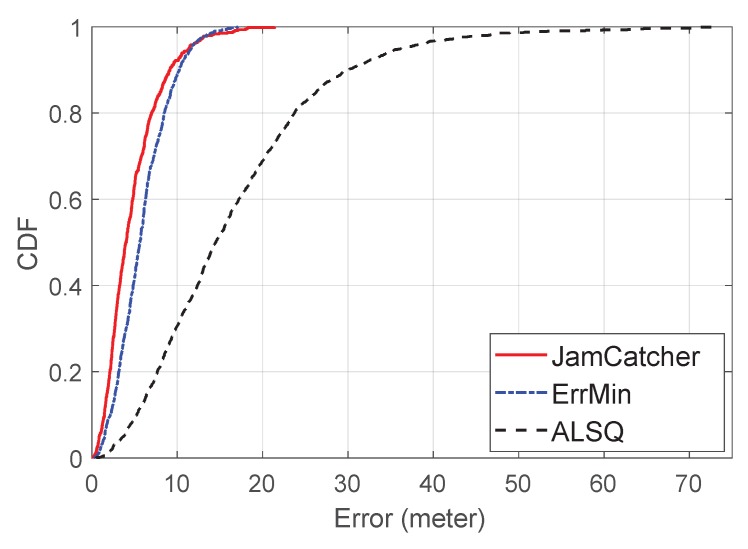
CDF of the localization errors of JamCatcher, ErrMin, and ALSQ.

The RMSEs of JamCatcher, ErrMin, and ALSQ were 5.36, 6.50, and 20.5 m, respectively. Figure 9 shows the CDFs of the localization errors of the three methods. We can see that JamCatcher achieved similar performance with ErrMin, and all of them outperformed ALSQ evidently. Note that although JamCatcher and ErrMin achieved similar performances, the number of observation nodes for JamCatcher (average of four nodes) was much less than that of ALSQ and ErrMin (average of 16 nodes). Thus, our proposed method is more effective and accurate with a limited number of observation nodes.

#### 5.2.3. Impact of Node Density

**Figure 10:**
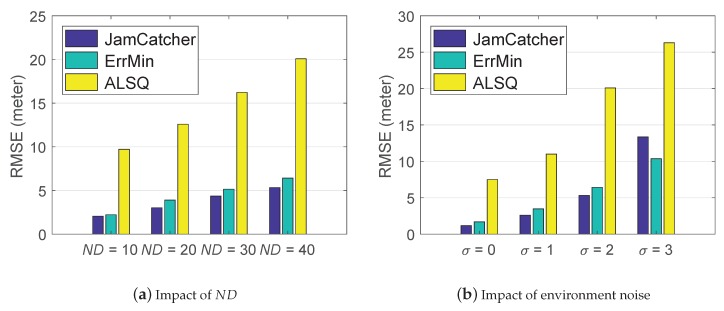
Impact of ND and σ on the performance of JamCatcher, ErrMin and ALSQ.

To evaluate the impact of node density, we changed the neighbor distance (ND) of the smart meters from 10 m to 40 m and calculated the RMSEs of the three algorithm. The results are shown in Figure 10a. When ND was set as 10 m, all three methods achieved their best performance, and the RMSEs of the three methods were 2.06, 2.20, and 9.74 m, respectively. As ND increased, the number of observation nodes decreased and the performance of each algorithm degraded, respectively. However, in every situation, JamCatcher and ErrMin achieved similar performance, and they always outperformed ALSQ.

#### 5.2.4. Impact of the Shadowing Effect

We set the value of the standard deviation of the random attenuation σ in the shadowing model from zero to three to evaluate the impact of the shadowing effect. Figure 10b shows the RMSEs of the three methods. The first observation is that when the σ was set as zero, which means no shadowing effect at all, the RMSEs of the three methods were still larger than zero, and the RMSE of ALSQ (7.9 m) was much larger than that of JamCatcher (0.42 m) and ErrMin (0.35 m). The reason is that estimation error of ALSQ had a positive correlation with the neighbor distance. As the neighbor distance in our simulation was large, i.e., 40 m, the estimation error of ALSQ reached 7.9 m even if there were no noise in the measurements. For ErrMin, it utilized a searching algorithm to search for the jammer’s location, by increasing the iteration times, the estimation error can converge to zero. As for JamCatcher, the estimation error exited due to the model error in our algorithm. The second observation was that as σ’s value increased, the RMSEs of the three methods increased, as expected. In any situation, JamCatcher and ErrMin achieved similar performance, and they all outperformed ALSQ.

#### 5.2.5. Tracking Performance in Different Routes

**Figure 11:**
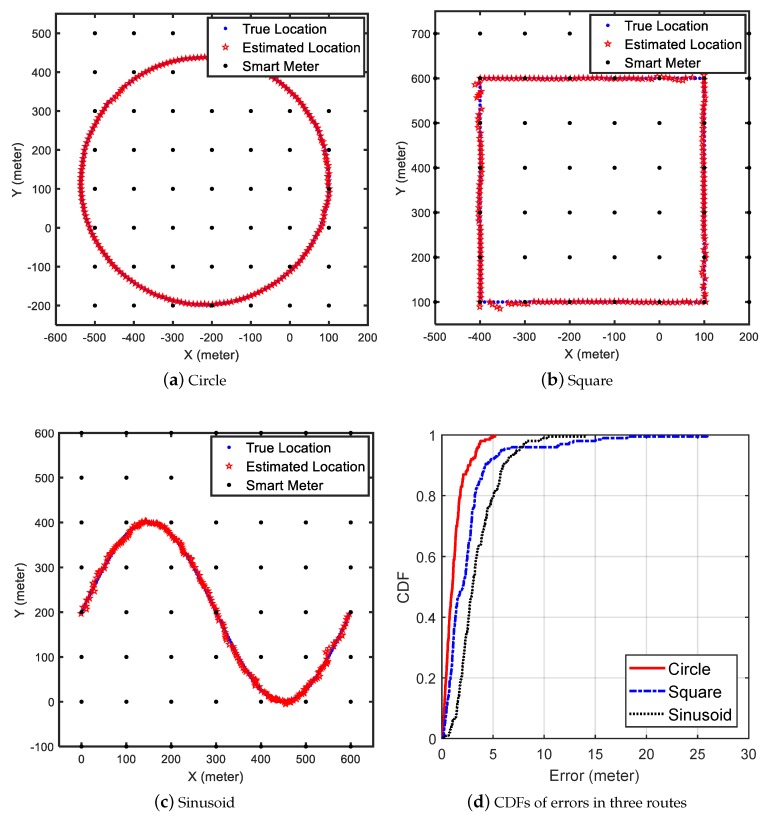
Simulation of three different trajectories the jammer moved along.

To evaluate the performance of JamCatcher in localizing jammers with different mobilities, we designed three moving routes for the jammer to move along (shown in Figure 11a–c). The first route was a circle, and the jammer moved with a constant velocity (10 m per second) and constant angular velocity (1.8 degrees per second). The second route was a square, where the jammer moved at a constant linear velocity (10 m per second) and zero angular velocity at each edge. The third route was a sinusoid, where the jammer moved with a changing linear velocity (maximum speed of 6 m per second) and changing angular velocity (maximum speed of four degrees per second). The neighbor distance ND of smart meters was 100 m. We set the path loss component η in the shadowing model as 2.11 and the standard deviation of the random attenuation σ to be 1.0. The maximum moving speed of the tracker was 20 m/s.

Figure 11d shows the CDFs of localization errors in three routes. The RMSEs of the three routes were 1.3, 4.0, and 4.6 m, respectively. We observed that JamCatcher performed better in the situation of Route 1, which was a circle. More than 90% of the estimation error was lower than 2 m. In the square route, the largest estimation error occurred in the four corners due to the sudden change in the direction of the jammer’s velocity. Among the three routes, JamCatcher performed worst in Route 3, since the speed of the jammer was changing frequently and the dynamic model in JamCatcher can only catch this change to some extent. Still, 90% of the estimation error was lower than about 6.5 m.

We changed the parameter of the shadowing model to see the impact of the shadowingeffects on JamCatcher, and Table 3 shows the results. In every condition, JamCatcher always performed best in Route 1, since the jammer’s velocity was stable. With the increase of σ’s value, the RMSEs of three routes increased by about 6 m. However, the RMSEs always stayed within 11 percent of the grid size, which was 100 m. When changing η to different values, we found that while the RMSEs of Route 1 and Route 2 were not sensitive to the inaccuracy in η (RMSEs changed by about 3 m), the RMSE of Route 3 changed evidently (from 9.87 m to 20.72 m). This was also caused by the frequent changing of the jammer’s speed.

#### 5.2.6. Capturing the Mobile Jammer

Based on the simulation setup in the three different routes, we also wanted to know how fast the tracker could catch the jammer and what was the minimum speed for the tracker. To do this, we defined a capture range of 10 m, and if the jammer stayed within a capture range longer than 10 s, we considered it a successful capture event. We defined the capture time as the time that the first capture event happens in the simulation. To find the minimum tracking speed, we set the tracker-jammer speed ratio value from 1.0 to 2.0. For example, in Route 1, the maximum speed of the jammer was 10 m/s; thus, we changed the speed of tracker from 10 m/s to 20 m/s. For each capture simulation, it stopped whether the simulation time reached 100 s or a capture event happened, and we repeated each simulation 100 times. We further defined a capture rate as the proportion of the times capture events happened within the 100 simulations.

**Figure 12:**
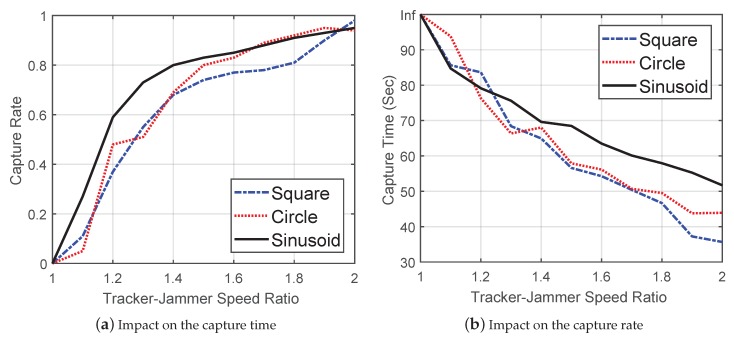
The impact of the tracker-jammer speed ratio.

The initial positions of the jammer in three routes were (100,100), (100,100), and (0,200), respectively. Since in each case, the tracker always started from (0,0), the initial distances between the jammer and tracker were 141, 141 and 200 m, respectively. Figure 12 shows how the tracker-jammer speed ratio affected the capture rate (Figure 12a) and average capture time (Figure 12b). When the value of the ratio was 1.0, the tracker was not able to catch the jammer since the distance would not decrease over time. As the speed ratio increased, the capture rate of all three routes increased and the capture time decreased. When the speed ratio was 2.0, the average capture time of the three routes was 35.66 s, 43.91 s, and 34.71 s, respectively. We also observed that the tracker’s speed did not have to be twice as much as the jammer’s speed; when the speed ratio was larger than 1.5, the capture rate of all three routes exceeded 75%.

## 6. Conclusions

With the development of UAVs, it will be possible to deploy a tracker (a drone or a mobile robot) in an AMI network to defend the smart meters from jamming attacks without human intervention. JamCatcher provides this kind of technology with a mobile jammer localization framework. JamCatcher divides the jammer localization process in AMI networks into two stages, i.e., the far-field chasing stage and near-field capturing stage, and provides algorithms for the corresponding stages. We evaluated algorithms in JamCatcher in both a real-world experiment and MATLAB simulations. The results show that JamCatcher outperformed existing jammer localization algorithms and could localize the mobile jammer effectively with limited observation nodes in a complex environment.

## Figures and Tables

**Table 1:** Estimation error (meter) comparison of all algorithms with different path loss exponents.

	GA	GPS	SA	ALSQ
η=2.00	0.23	0.23	0.23	0.65
η=2.25	0.21	0.20	0.20	0.65
η=2.50	0.24	0.22	0.21	0.66
η=2.75	0.27	0.24	0.25	0.67
η=3.00	0.31	0.29	0.29	0.68

**Table 2:** Definition of the parameters in the simulation.

Parameter	Meaning	Value
*L*	Size of AMI Network	500 m
ND	Neighbor Distance	40 m
$P_{J}$	Transmit Power of Jammer	100 mW
$P_{T}$	Transmit Power of Smart Meter	50 mW
*G*	Gain of the Antenna	1
$P_{N}$	Power of Ambient Noise	−65 dBm
$f_{T}$	Signal Frequency	2.4 GHz
η	Path Loss Exponent	2.40
$\gamma_{0}$	SNR Threshold	−3 dB
$d_{T}$	Tracker’s Communication Range	60 m
$d_{J}$	Jammer’s Jamming Range	80 m

**Table 3:** RMSE (meters) in the three routes with different noise levels and different path loss exponents.

Route	σ					η				
	0	1	2	3		2.00	2.25	2.50	2.75	3.00
**1**	0.66	1.90	3.41	5.38		2.21	2.32	3.17	4.16	5.35
**2**	4.78	5.43	8.10	10.92		5.82	6.03	6.48	7.12	7.40
**3**	3.52	4.79	8.28	10.77		9.87	9.94	16.15	19.46	20.72
